# Congenital infantile digital fibromatosis: a case report and review of the literature

**DOI:** 10.4314/ahs.v20i4.42

**Published:** 2020-12

**Authors:** Omolade O Adegoke, Akinlabi E Ajao, Gbemi H Ano-Edward

**Affiliations:** 1 University of Ibadan College of Medicine, Pathology; University College Hospital Ibadan, Pathology; 2 Bowen University, Department of Surgery; University College Hospital Ibadan, Department of Surgery; 3 Bowen University Teaching Hospital, Anatomic pathology; Bowen University, Department of Anatomic Pathology

**Keywords:** Fibromatosis, digits, inclusion body

## Abstract

Infantile digital fibromatosis (IDF), also called inclusion body fibromatosis is an uncommon benign tumour occurring in the digits of young children. In about a third of cases, it is congenital and the diagnosis is based on the presence of peculiar intracytoplasmic inclusions on histology. Recurrence rate post-surgery is high. However, spontaneous regression has been reported. We present a case of a 5-month-old infant who had excision of a right second toe mass, which has been present from birth. Histological examination revealed this to be infantile digital fibromatosis. To the best of our knowledge, no report of this has been made in Nigeria. It is important that this diagnosis be entertained in young children with masses on the digits as this will influence the management instituted.

## Introduction

Infantile digital fibromatosis is an uncommon fibroblastic and myofibroblastic tumour occurring in the digits of young children.^1^ This benign tumour commonly occurs in the dorsolateral aspects of toes and fingers excluding the thumb and big toes.^1^,^2^ Patients mostly present in the first two years of life and about a third of cases are congenital.^3^ Intracytoplasmic inclusions in the fibroblastic cells are characteristic features in this tumours^1^–^3^. Recurrence rate post-surgery is high and spontaneous involution has been described in some cases.^1^ A knowledge of the possible differentials of finger and toe masses is essential for proper management of these cases. To the best of our knowledge, this tumour is not well known in Nigeria and Africa.

## Case report

**Figure 1:**
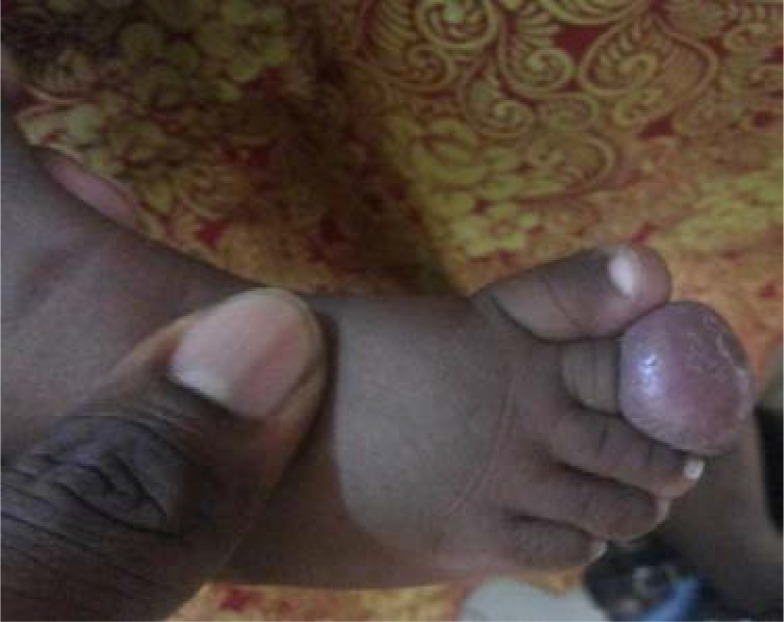
showing soft tissue mass on the second toe.

The patient is a 5-month-old female infant who presented with a painless round swelling on the second toe of the right foot, which had been present from birth. The swelling was located on the dorsum of the toe proximal to the nail and gradually increased in size to obliterate the nail bed. The parents brought the child to our facility on account of increasing size of the mass. Physical examination revealed a pedunculated, non-tender soft tissue mass, measuring 4.5 x 3 cm, with the base located on the middle phalanx of the right second toe, spreading over the nail bed (Figure 1). Radiological evaluation revealed a soft tissue mass over the right second toe with no bony involvement.

The mass was excised with primary closure of the wound. There was initial primary hemorrhage which was controlled with pressure dressing and the child was subsequently discharged from the hospital. The wound healed satisfactorily without any adverse sequelae.

At the histopathology laboratory, we received a piece of cystic tissue measuring 2x2x1 cm. Sectioning, revealed greyish white solid tissue and an empty cystic cavity. Microscopic examination revealed skin tissue with intact epidermis, proliferating fibroblasts arranged in bundles, forming a poorly defined nodule within the dermis on Haematoxylin and Eosin (H&E) stain. Also seen were characteristic perinuclear eosinophilic inclusions on high power (Figure 2).

**Figure:**
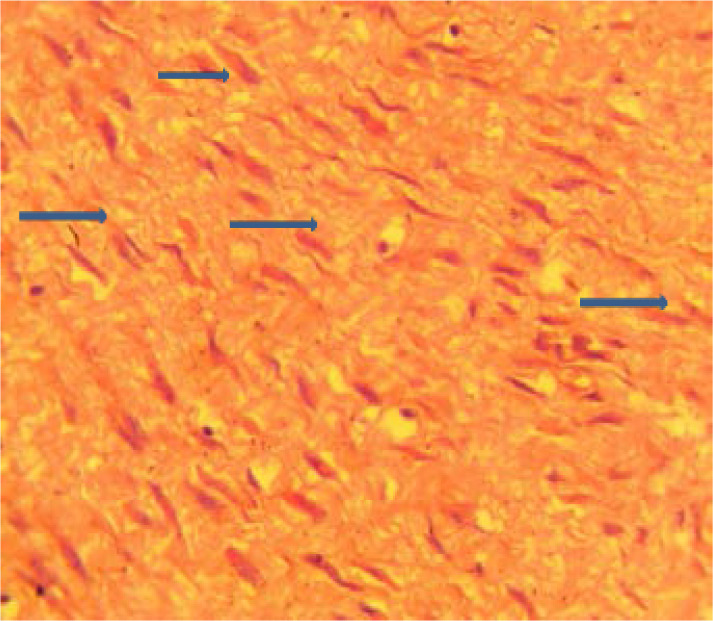


Masson's trichrome (MT) stained the intracytoplasmic inclusions red (Figure 3). The histologic findings were in keeping with infantile digital fibromatosis also called inclusion body fibromatosis.

**Figure:**
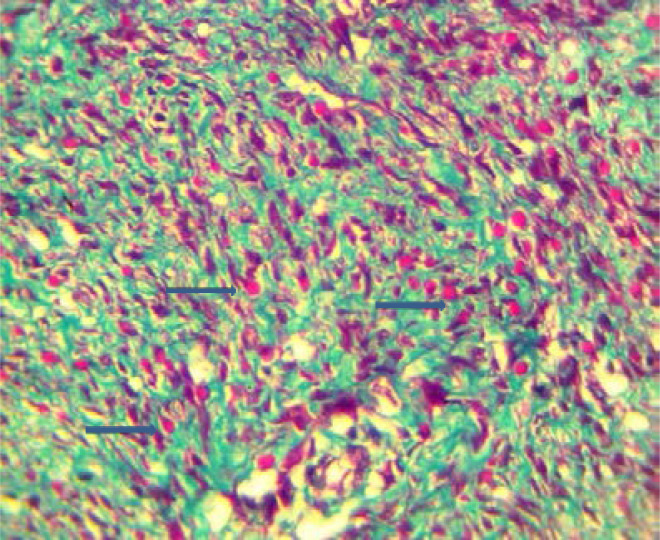


## Discussion

Infantile digital fibromatosis is also called Reye's Tumour and inclusion body fibromatosis. It is a benign tumour first extensively described by Reye as recurring digital fibrous tumour in 1965.^2^,^4^ It occurs predominantly in digits, has a high recurrence rate and is characterised by intracytoplasmic inclusions on histology.^1^–^3^ The aetiology of inclusion fibromatosis is unknown. A viral aetiology was initially suspected because of the presence of cytoplasmic inclusions but polymerase chain reaction and electron microscopy have ruled out this possibility.^2^,^4^,^5^,^6^ Inclusions are now known to be composed of densely packed vimentin and actin filaments and is thought to arise due to aberrant assemblage of microfilaments in myofibroblasts and smooth muscle cells.^7^

The tumour may be single or multiple, firm or gelatinous in consistency^1^–^7^. Nodules occur in the dorsolateral aspects of toes and fingers excluding the thumb and big toe. Although, tumours with similar appearance have been described in extra digital sites such as the breasts and thorax;^1^,^8^,^9^ the fingers are more often involved than the toes^10^ and rarely it may be associated with pain or functional impairment.^2^,^7^

About a third of cases are congenital as occurred in this patient, while most will present within the first year of life. A few cases have been described in adults. There is no sex predilection^11^ and no suggestion that antecedent trauma may influence its development. It accounts for about 0.1% of fibrous tumours in children.^3^

Radiographs show a non-specific soft tissue mass, the underlying bone may be involved with either erosion or invasion but only rarely^10^. The tumour is benign, although recurrence following surgery is high with rates of up to 60%.^12^ Recurrence occurs at the same site and sometimes a second tumour may develop in an adjacent finger or toe.^13^

Histological features are unique, interdigitating fascicles of spindle cells and collagen fibres make up the tumour which is located in the dermis and may extend into the subcutaneous tissue.^2^–^4^ Intracytoplasmic inclusions can be seen on H&E at high power.^1^–^6^ Histochemical stains are useful in making a diagnosis.^2^ The cytoplasmic inclusions are eosinophilic with Masson's Trichrome stain, which was used for this case. The inclusions also stain deep purple with phosphotungstic acid hematoxylin (PTAH), yellow with elastic van Gieson, and bright red with Lendrum's phloxine-tartrazine,^2^ while it is negative with Periodic acid Schiff(PAS).^9^ The tumour may occasionally involve the periosteum and erode the bone^14^. It is important that an exhaustive search be made for inclusion bodies when this diagnosis is suspected. The inclusions are sometimes difficult to see on the conventional H&E, however, utilising readily available histochemical stains such as Masson's trichrome will make it easier. Immunohistochemistry is positive for vimentin, cytokeratin, desmin and muscle-specific actin, suggesting a myofibroblastic origin, while it is negative for S-100 protein and glial fibrillary acidic protein, and is reported to be positive for CD57 in some cases.^15^ Electron microscopy shows the cells contain bundles of microfilaments running longitudinally within the cell. The inclusion bodies are made of densely packed fibrils similar to those in the long bundles. These ultrastructural features are in keeping with a myofibroblastic origin.^2^,^13^,^15^

Due to the benign nature of the tumour, recent studies recommend a conservative management with regular monitoring.^16^ This was not done in this case as there was an increase in the size of the tumour and diagnosis was made after surgery. Waiting for spontaneous regression might be a little difficult in this environment as attendance at follow up clinics is notoriously poor and patients would often seek traditional medical practitioners who may further complicate the lesion. Cases with spontaneous involution have been reported in literature, it usually occurs within 2 to 3 years.^17^ Other reasons to consider in conservative approach are the highrate of recurrence and the possible occurrence of deformities in the digits following surgery.^16^,^17^,^18^ Surgery is indicated where there is deformity and functional impairment..^8^ In this case, patient was brought to the hospital when there was an increase in size of the tumour. Topical corticosteroids have shown no benefit although, intra-lesional corticosteroids might be beneficial. Other proposed treatments include intra-lesional fluorouracil, which though painful, give very good response without recurrence.^19^ Mohs micrographic resection has also been used with good outcome.^7^,^20^ The differential diagnosis of IDF includes keloids, hypertrophic scar tissue, terminal osseous dysplasia and pigmentary defects, and juvenile aponeurotic fibroma.^2^,^7^

Complications are rare and include deformity, functional impairment and ulceration7. Spontaneous regression without scarring occurs, although the exact rate is not known.^21^

## Conclusion

Congenital Infantile digital fibromatosis is a rare benign tumour occurring predominantly in infants. Preoperative diagnosis is uncommon because it is often not considered in the differential diagnosis of nodular digital tumours in children. It is important for surgeons to consider it as a diagnosis as the current management of IDF is non-surgical. However, in this environment, surgical management is advised because patients often end up with untrained caregivers and quacks that will worsen the condition when left untreated.
